# Lung Cancer Diagnoses and Outcomes During the Syrian War, 2011-2018

**DOI:** 10.1001/jamanetworkopen.2024.2091

**Published:** 2024-03-13

**Authors:** Ibrahem Hanafi, Dana Abo Samra, Rama Alsaqqa, Ahmad Naeem, Baraa Shebli, Ghassan Ajlyakin

**Affiliations:** 1 Division of Neurology, Department of Internal Medicine, Faculty of Medicine, Damascus University, Damascus, Syria; 2 Department of Oncology, Faculty of Medicine, Damascus University, Damascus, Syria; 3 Faculty of Medicine, Damascus University, Damascus, Syria; 4 Division of Cardiology, Department of Internal Medicine, Faculty of Medicine, University of Aleppo, Aleppo, Syria

## Abstract

**Importance:**

Protracted wars, which disproportionately affect low-resource countries, exacerbate the challenges faced by cancer care systems, with lung cancer being the most affected as the most fatal oncological condition.

**Objective:**

To characterize the presentation and survival of patients with lung cancer during the decade-long Syrian war.

**Design, Setting, and Participants:**

This cohort study included patients at a large oncology center in Syria during the first 8 years of the Syrian armed conflict (2011-2018). All patients with a new diagnosis of lung cancer were included. Variables investigated included histological subtypes, TNM classification (tumor, lymph nodes, and metastasis), and staging at diagnosis as well as the yearly follow-ups up to 5 years after presentation.

**Exposure:**

The Syrian war divided the country into temporary regions with different political influences and heterogeneously impacted health care accessibility among these regions.

**Main Outcomes and Measures:**

Change in prevalence of advanced lung cancer cases at presentation; change in overall survival odds.

**Results:**

The study included 5160 patients from all Syrian governorates (mean [SD] age, 59.6 [10.8] years; 4399 men [85.3%]). New diagnoses sharply declined during the first 3 years of the war, with partial recovery afterward. Regardless of their tempo-geographical regions, 60% to 80% of the yearly diagnoses presented with metastases. The 1-year and 5-year survival rates were 13.1% (423 of 3238 patients with follow-up results) and 0.1% (2 of 1853 patients), respectively. Patients who presented from regions more involved in the armed conflicts showed poorer survival rates with odds ranging between 0.51 (95% CI, 0.44-0.59) and 0.61 (95% CI, 0.49-0.74) across follow-ups for up to 2 years in comparison with patients presenting from safer regions. War-related health care inaccessibility explained a greater percentage of the variability in survival (Nagelkerke R^2^ = 7.5%; *P* < .001) than both patients’ age and the stage of the disease combined (Nagelkerke R^2^ = 3.9%; *P* < .001).

**Conclusions and Relevance:**

The Syrian war has been associated with a marked decline in the management of patients with lung cancer, with limited access to specialized care, delayed diagnoses, and substantial decrease in survival rates correlating with the intensity of armed conflict in the respective regions. The profound repercussions of the prolonged armed conflict on patients with lung cancer in Syria necessitates urgent comprehensive strategies to improve the accessibility and quality of health care services, especially in conflict-ridden zones.

## Key Points

**Question:**

Is the Syrian war associated with changes in lung cancer diagnoses and outcomes?

**Findings:**

This cohort study of 5160 Syrian patients found a 0.1% 5-year survival rate for patients with lung cancer diagnosed during the war, with 80% of patients presenting with metastases. Survival odds were notably lower for individuals presenting from zones heavily impacted by the armed conflicts compared with those presenting from relatively safer areas.

**Meaning:**

These results suggest that during the first 8 years of the Syrian war, exposure to the conflict was associated with poor accessibility to cancer care, the presentation of patients with more advanced cancer stages, and, subsequently, extremely low survival probabilities, correlating with the gravity of the armed conflicts in the region of residence.

## Teaser

This cohort study examines the association of exposure to the Syrian war from 2011 to 2018 with stage of diagnosis and survival odds among patients with lung cancer.

## Introduction

The number of people diagnosed with lung cancer globally ranks second after breast cancer. However, lung cancer tops the list of killing cancers in men and women worldwide.^1^ It is one the most serious emerging diseases of the 21st century^2,3^ and currently attracts major public health attention, because it is projected to kill approximately 3 million people in 2035.^4^ The incidence rates of lung cancer vary geographically and chronologically, which can only emphasize the unmet need to comprehensively report the dynamics of its epidemiology worldwide.^4,5^ One of the main challenges toward a worldwide up-to-date epidemiological registry is that more than half of the world population are covered by neither cancer registries nor death certification systems.^4^ This is mostly evident in low- and middle-income countries, which fall significantly behind developed health systems in lung cancer care due to belated diagnoses, limited diagnostic tools, and a severe shortage of experienced physicians.^6^

Wars, which disproportionately prevail in low-resource countries, have an unparalleled impact on cancer care.^7,8^ In conflict zones, a multitude of factors, such as the destruction of health care facilities, restricted access to care, medication shortages, and limited diagnostic resources collectively contribute to exaggerated cancer mortality rates.^9^ In the case of the Syrian conflict, the 12-year war has evolved into one of the most severe global humanitarian crises, displacing large masses of people and leading the Syrian population, initially around 23 million in 2011 (reported pre-war), to fall below 19 million by 2016.^10^ More than 12 million are in dire need of health care, as the war also forced about 70% of health workers to flee Syria and excluded half of the primary health facilities from service,^11^ affecting the management of both communicable and uncommunicable diseases.^12,13,14,15,16,17,18,19^ Furthermore, diagnostic and therapeutic approaches became progressively restricted, often proving prohibitively expensive for patients to access.^8,17^

In the realm of cancer care, approximately 18 000 new cases were diagnosed in 2008, including 1818 cases of lung cancer.^20^ This number increased to almost 21 000 in 2020, encompassing 1976 patients with lung cancer.^21^ Meanwhile, Al-Kindi Hospital, formerly the largest hospital in northern Syria, was entirely destroyed during the conflict, compelling most patients with cancer throughout the country to converge on one major center in Damascus for specialized cancer care.^22,23^ Accessibility to this center still faced constant disruptions due to escalating armed conflicts that fragmented the country into territories with varying political influences. These circumstances compounded the challenges in registering, diagnosing, reporting, and managing patients with cancer,^22,24,25^ and the consequences of Syrian war on the prognosis in patients with lung cancer, the most lethal oncological condition, remains unexplored. In this cohort study, we aim to investigate how this protracted armed conflict was associated with the presentation and outcome in Syrian patients with lung cancer.

## Methods

### Study Design and Patients

This is a retrospective cohort study performed at the lung cancer unit of Al-Beiruni Cancer Center in Syria, which became the main cancer center in the country after the destruction of Al-Kindi Oncology Center in Aleppo in 2016^22^ and has since been receiving about 60% of all patients with cancer in Syria (around 11 000 cases per year).^23^ This Damascus University–affiliated public hospital provides free-of-charge care to patients from across the country, including medical consultancy, medications, chemotherapy, radiotherapy, surgeries, and hospital and intensive care stays. However, like the rest of the country, this center experienced scarcity of oncologists during the war, as the best ratio was in Damascus with 20 oncologists for a population exceeding a million. Moreover, only fundamental diagnostic tools such as computed tomography (CT) scans and biopsies were available, restricting the ability to explore potential war-related exposures, such as radon and mustard gases. Nevertheless, this center maintained accessibility to surgeries as well as chemotherapy and radiotherapy throughout most of the study period, unlike most of the other Syrian cities.^8^ Chemotherapy and radiotherapy were administered according to the most recent guidelines but were in many cases constrained by drug availability.

Our cohort included patients who received a diagnosis of primary lung cancer between 2011 and 2018, a period that spanned over the first 8 years of the Syrian war. Although the financial collapse and humanitarian crisis persist to date, most of the military actions, sieges, and displacement happened during the included period. In April 2021, the ethical approval was obtained from the institutional review board at the faculty of medicine of Damascus University, which waived the need for individual informed consent from the patients included in the study because it involved no risk, required no identifiable information, and was not practicably feasible to conduct retrospectively with consent. Reporting of this study followed the Strengthening the Reporting of Observational Studies in Epidemiology (STROBE) reporting guideline.

#### Data Collection

The medical archives of the included patients were scanned by a group of 10 senior medical students or recent medical graduates at Damascus University. They were all masked to the study hypothesis and used a standardized data extraction questionnaire with explicitly predefined variables for data extraction. Each patient’s medical records were screened by 1 data collaborator. In cases of ambiguity or uncertainty, the specialized oncologist (D.A.S.), who closely oversaw data collaborators, was consulted. The extracted data included demographic variables, pathological diagnosis, and yearly follow-ups for up to 5 years. Each of these follow-ups was documented by the specialized oncologists following the patients based on clinical examination and CT scans and according to the new response evaluation criteria in solid tumors (RECIST) updated in 2009.^26^ We also collected the staging report and TNM (tumor, lymph nodes, and metastasis) classification of the tumor according to the 7th edition of the American Joint Committee on Cancer (AJCC) criteria for lung cancer.^27^

**Figure 1.**
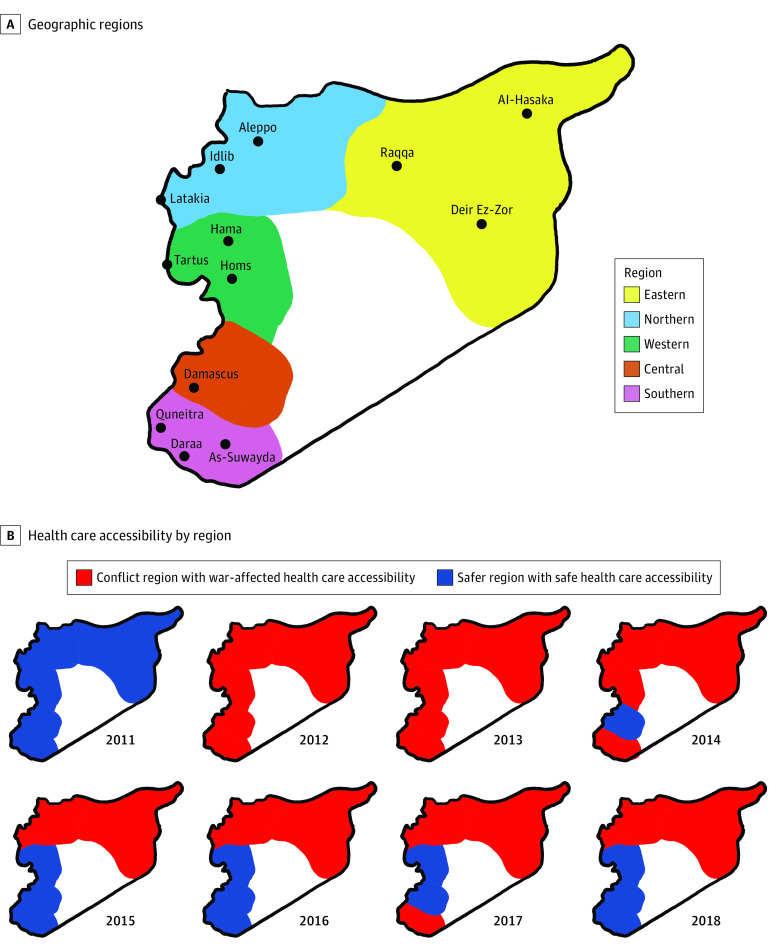
Progression of War-Related Health Care Inaccessibility in Syria’s Geographic Regions Over the Years of War

Collected information about place of residence at diagnosis was analyzed in detail, as it indicated information about the dynamics of the Syrian war and its impact on the diagnosis and survival. The regions of geopolitical control in Syria changed over the years of the Syrian armed conflict, which caused dramatic changes in health care accessibility and the ability for patients to present to our center from most cities.^28^ Therefore, we grouped the 14 Syrian governorates into 5 geographic regions. Furthermore, we used the dynamic map of geopolitical influence over the study years to group the regions temporally into conflict zones and safer zones.^28^ All regions were grouped as safe during 2011 as this year witnessed a small amount of localized armed conflicts that did not affect mobility around the country. In contrast, all regions were considered conflict areas in 2012-2013 because the armed conflicts widened and were spontaneously involving different cities before the appearance of areas with different geopolitical influences. Toward the end of the study period, the situation eased in central Syria and the safe areas widened south and west. In contrast, the northern and eastern regions remained under the conflict classification as patients’ mobility from these areas to Damascus stayed almost always dangerous until 2018 (Figure 1).

### Statistical Analysis

We analyzed the data using the Statistical Package for the Social Sciences version 23.0 (SPSS Inc) and created the figures using Excel 365 version 2011 (year 2020) (Microsoft) and Illustrator version CS6 (Adobe). Due to the high loss of follow-ups, survival was presented as percentages calculated only for the patients who managed to come to the hospital at the corresponding follow-up. A χ^2^ test was used together with odds ratios (ORs) to investigate the association between categorical variables. Binary logistic regression analysis was also used to explain the variability in 1-year survival using patients’ age and disease stage as well as the interaction variable between the geographical region and year. An α value of 0.001 was used to determine the level of statistical significance.

## Results

Our inclusion criteria were met by 5160 patients with lung cancer; 4399 (85.3%) were males, with a mean (SD) age of 59.9 (10.5) years for men and 57.8 (12.1) for women (*P* < .001). The 14 Syrian governates were represented in our sample and the highest proportions of patients resided in Damascus (793 [15.4%]), Homs (609 [11.8%]), rural Damascus (592 [11.5%]), and Aleppo (572 [11.1%]). The most common comorbidities in the sample were diabetes (279 [5.4%]) and hypertension (301 [5.8%]). By gender, 3462 men (92.8%) and 287 women (47.0%), respectively, were smokers (*P* < .001); the mean (SD) pack-year value was 56.5 (32.8).

**Figure 2.**
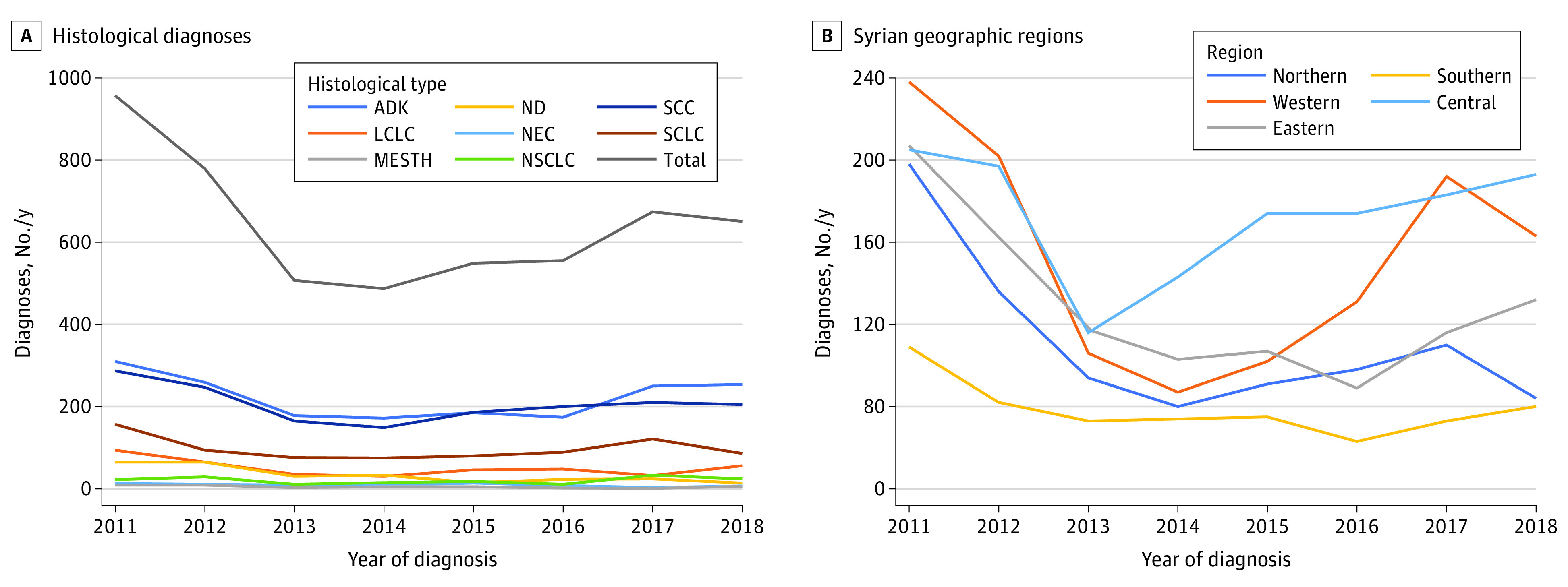
Evolution of Lung Cancer Cases Throughout Syrian Armed Conflict ADK indicates adenocarcinoma; LCLC, large cell lung cancer; MESTH, mesothelioma; NEC, neuroendocrine carcinoma; ND, unspecified lung cancer; NSCLC, non-small cell lung cancer; SCC, squamous cell carcinoma; SCLC, small cell lung cancer.

The 2 most common histological diagnoses were adenocarcinoma (1782 cases [34.5%]) and squamous cell carcinoma (1649 cases [32.0%]). The proportions of the histological subtypes remained stable throughout the 8 studied years. However, the number of cases dramatically decreased from approximately 1000 cases in 2011 to less than half that in 2014. This drop was followed by a steady slow increase in the number of cases toward 2018 (652 cases). A closer look into the geographical dynamics of these changes showed that the decrease of the number of cases involved all Syrian governorates. However, the following rise was focused in central, western, and southern Syria, which nearly restored their rates of 2011. In contrast, this retrieval did not take place in northern and eastern areas of the country, whose rates stabilized at around 50% of their numbers in 2011 (Figure 2). It is worth mentioning that prevalence by age and gender were relatively similar when compared across the investigated years.

**Figure 3.**
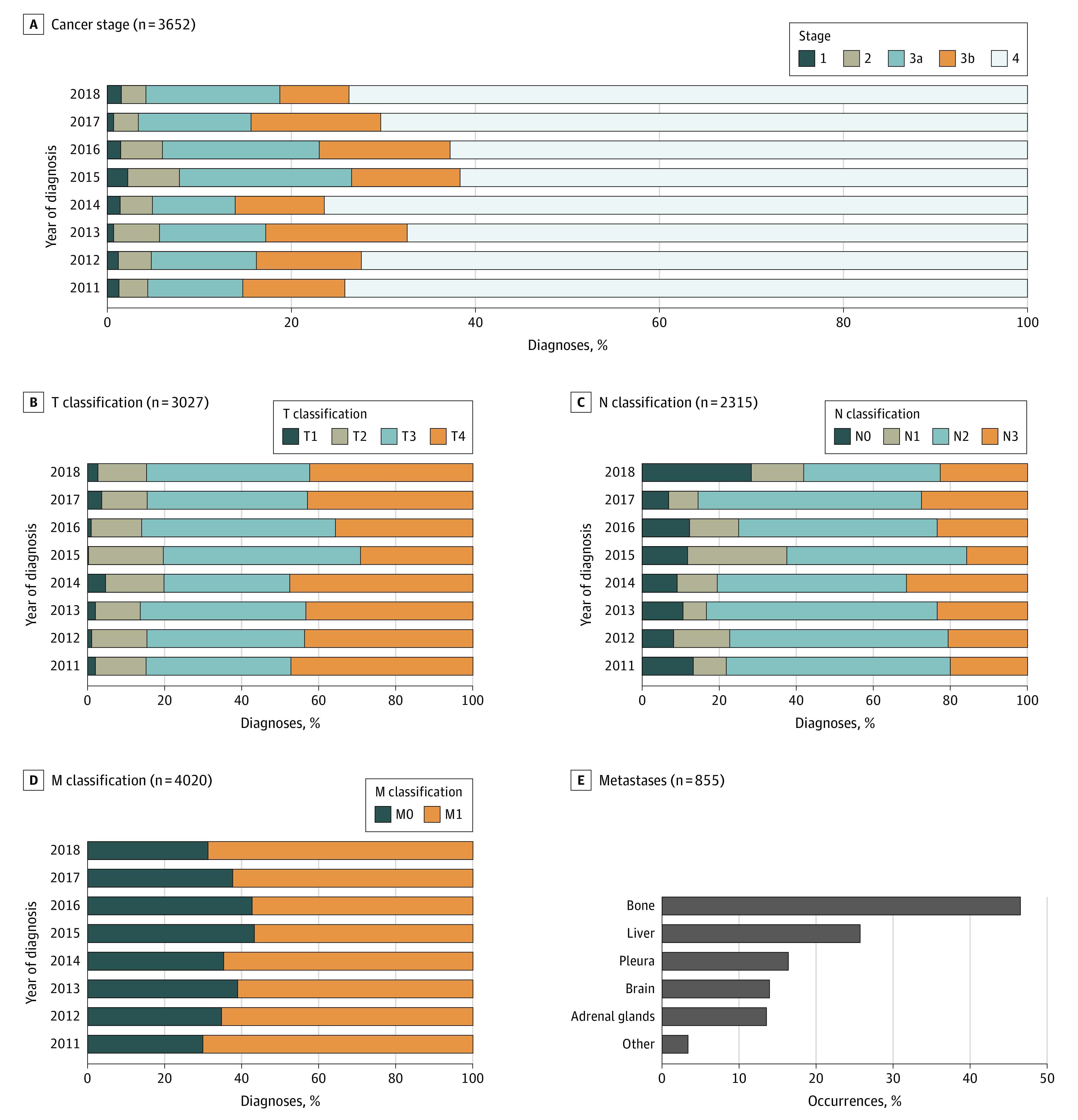
Stages, TNM Classifications, and Metastases of Lung Cancer Cases During the Syrian War TNM indicates tumor, lymph node, and metastasis classifictions.

Our study showed that 60% to 80% of the Syrian lung cancer cases in each year of the study period received their diagnosis with stage IV, without stage migration across the study years. In the meantime, less than 10% of our patients were diagnosed with stage I or II in all the studied years, without significant differences between the regions in terms of staging. Metastases were discovered in 2558 patients (63.6%) at diagnosis, and the most common metastases were to bones (398 [39%]) and liver (220 [21%]) (Figure 3).

**Figure 4.**
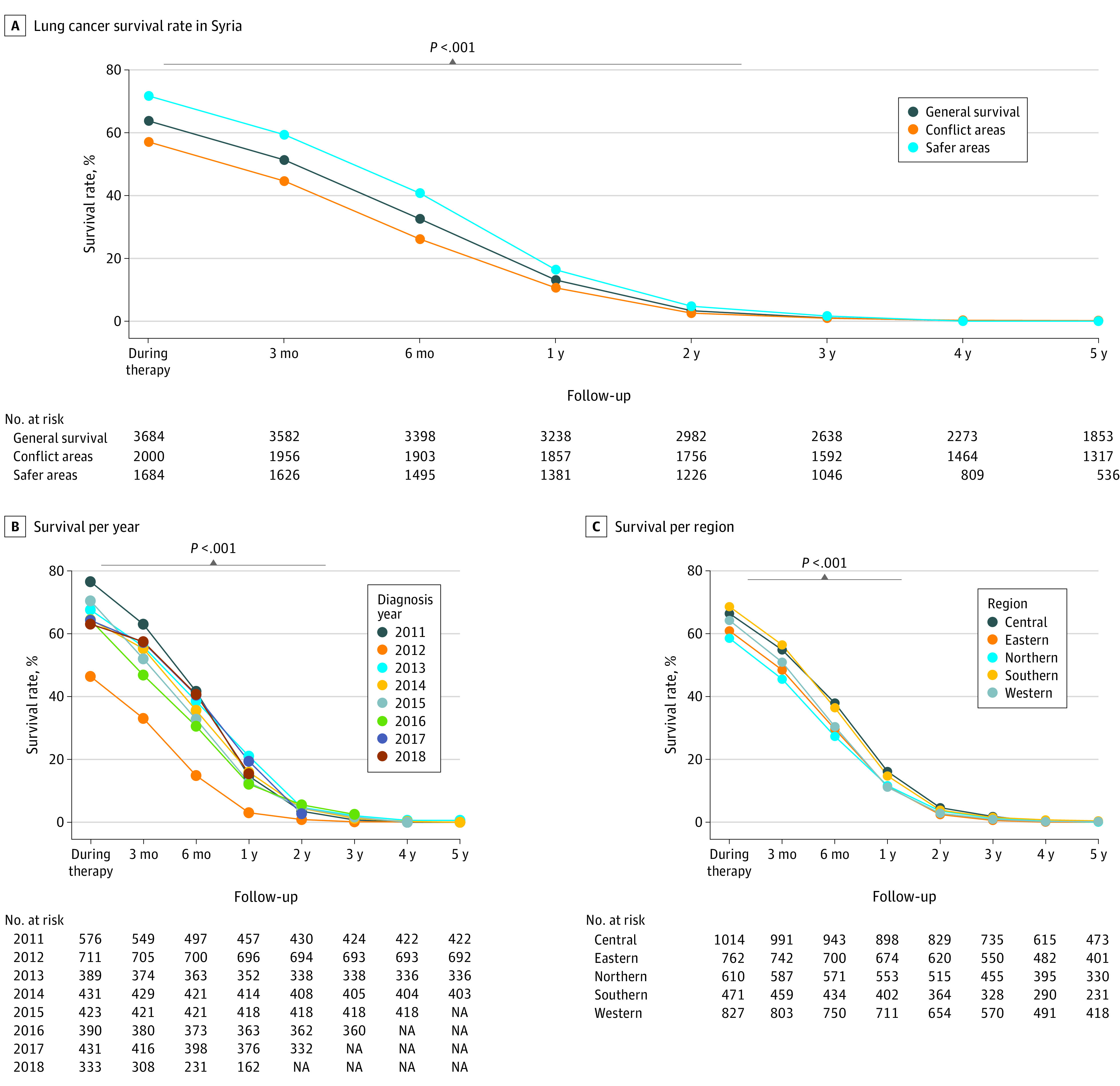
Survival Rate Comparisons Among Patients With Lung Cancer Across Different Syrian War–Related Tempo-Geographical Regions Panel A, the gray bar indicates differences in the survival rate between conflict and safe areas that were statistically significant; panels B and C, differences among corresponding groups that were significant (*P* < .001).

Follow-up reports were available for 4192 patients, which represented 81.2% of our complete sample. A total of 508 cases were lost to follow-up during the first course of therapy, and this number steadily grew during the first year of follow-up to reach 954 patients. This increase in the number of lost cases coincided with the drop in survival during the first year after diagnosis (eFigure in Supplement 1). The survival rate during therapy was 2348 patients (63.7%), which dropped to 423 patients (13.1%) at 1 year and 2 patients (0.1%) at 5 years, without significant differences among the 3 most common histological types (ie, adenocarcinoma, squamous cell carcinoma, and small cell lung cancer). Patients diagnosed during 2012 had significantly poorer survival in comparison with cases diagnosed in all the other years (1-year survival: OR, 0.17; 95% CI, 0.11-0.26; *P* < .001). Similarly, the survival rates differed significantly between geographic regions with the poorest outcomes for northern and eastern Syria (1-year survival vs rest of country: OR, 0.79; 95% CI, 0.63-0.98; *P* < .03). Finally, grouping the cases as presenting from conflict areas vs safe areas revealed significantly poorer outcomes for the first 2 years in regions that were more affected by the Syrian armed conflict (Figure 1). Compared with safe regions, the OR of survival when presenting from conflict regions was 0.52 (95% CI, 0.46-0.60; *P* < .001) during the first course of therapy and remained between 0.51 (95% CI, 0.44-0.59) and 0.61 (95% CI, 0.49-0.74) for the following follow-ups up to 2 years from diagnosis (Figure 4).

**Figure 5.**
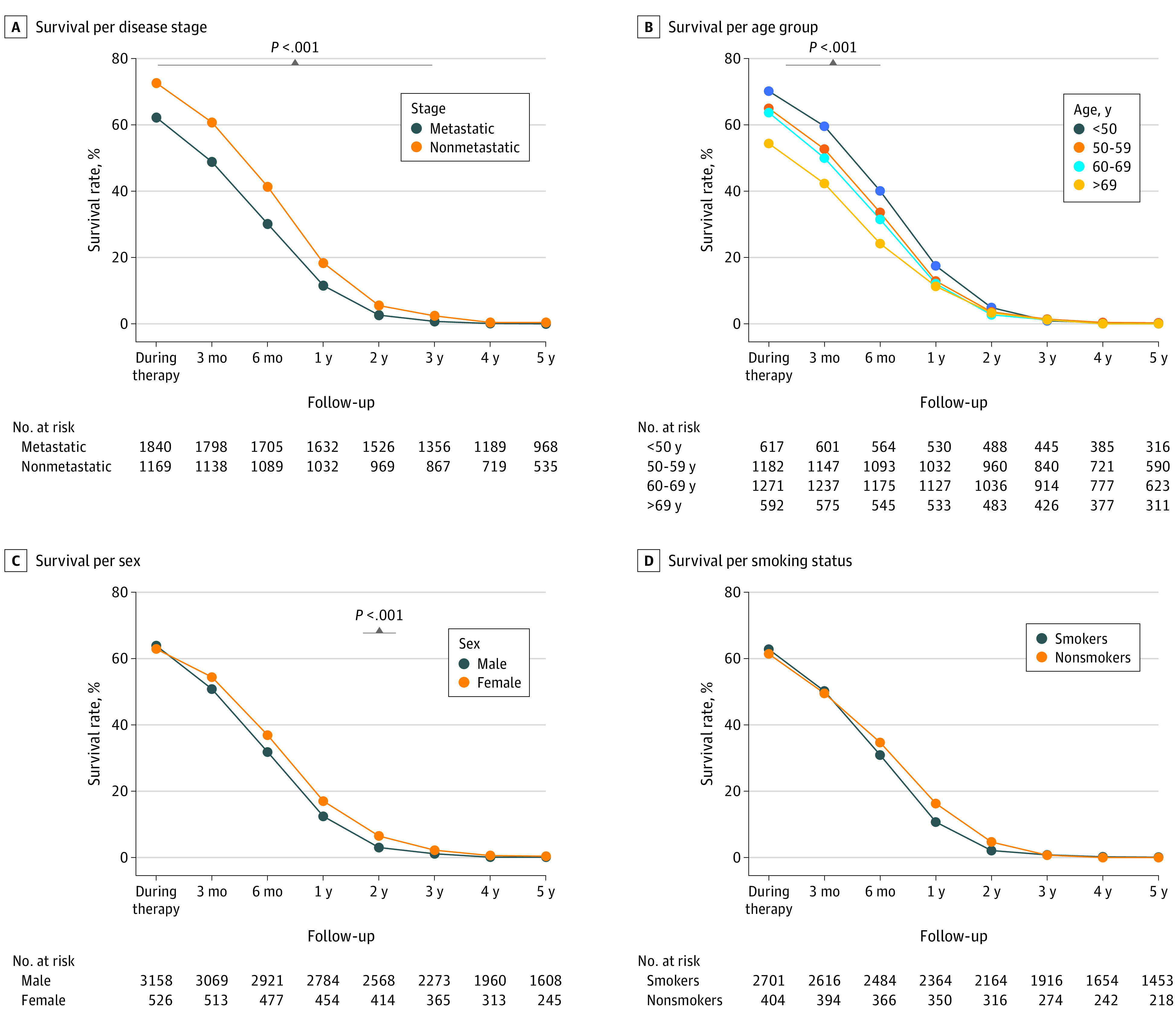
Survival Rate Comparisons Among Syrian Patients With Lung Cancer Across Various Demographic Factors The gray bar above panels A through C indicates differences in the survival rate among the corresponding groups that were statistically significant (*P* < .001).

Similarly, survival was compromised in patients with metastatic lung cancer (1-year survival: OR, 0.58; 95% CI, 0.46-0.72) or advanced age of 50 years and older (1-year survival: OR, 0.65; 95% CI, 0.51-0.84), with no perceptible impact from gender or smoking status (Figure 5). Therefore, we performed a binary logistic regression analysis to study the independent variables contributing to the first-year survival variability (as a dependent variable) and to investigate the association of the Syrian war (ie, interaction between geographic region and diagnosis year) with the fatality of the disease. The first block of independent variables included disease stage and age at diagnosis producing a significant model but with low performance (Nagelkerke R^2^ = 3.9%; *P* < .001) (eTable 1 in Supplement 1). Adding the interaction variable between the geographic region and the diagnosis year as a secondary block to the regression improved the performance of the model significantly (Nagelkerke R^2^ = 12.2%; *P* < .001), indicating that the war-related health care inaccessibility explained more of the variability in survival than both the staging and age combined (eTable 2 in Supplement 1).

## Discussion

As the first cohort study, to our knowledge, on patients with lung cancer in Syria, our study succeeded in characterizing the presentation and survival in people with lung cancer during the protracted Syrian armed conflict. Our data first revealed a generalized steep decrease in the number of new cases during the first few years of the war, followed by partial recovery distinctively in the regions where the armed conflicts eased. Our findings also suggested that the diagnosis of patients with lung cancer during the Syrian war was delayed, as 63.6% of patients presented with metastatic cancer. Furthermore, the war was associated with extremely low survival rates, with significantly poorer outcomes for patients presenting from more-impacted conflict zones.

The number of people diagnosed with lung cancer at our center halved during the first 2 to 3 years of the war, and this can be attributed to several war consequences including the nationwide displacement of population with almost 6 million registered refugees by the end of 2018,^29^ the inaccessibility to primary health care, and the insecurity to commute across the country for secondary care.^8,17,30^ This can also justify that the number of cases completely recovered afterwards in stabilizing regions as the armed conflicts reduced; a tendency that was less clear in the northern and eastern areas of the country, where significant insecurity persisted with the formation of new political influences.^31^ In contrast, several studies reported higher incidence of lung cancer in people who witnessed wars like in Iran,^32^ Vietnam,^33^ and Italy.^34^ Nevertheless, it should be noted that such studies reported the status of lung cancer in the postwar period, while the pattern we present in this study better illustrates the acute consequences of war, which disallowed some patients from receiving the care offered at other parts of the country, rather than long-term changes in the incidence rate.

Distant metastases were identified in 63.6% of our patients at presentation. This percentage is remarkably higher than the 53% reported in the US.^35^ Although the lack of reports from Syria before the war obscures the baseline status of lung cancer presentations, direct and indirect links can be drawn between the current status and war repercussions. We found with a comparable sample of patients with breast cancer that the war was significantly associated with a prolonged period between noticing the symptoms and diagnosis, leading to higher stages at presentation.^36^ Additionally, several reports described how the Syrian armed conflict affected the availability of specialized physicians, health care facilities, and screening programs, and increased the challenges in cancer diagnosis.^8,37^ It should be noted that this delay in diagnosis also complicates the management plans and poses higher financial burden on the patients and the system.^38^

Our study also showed a 5-year survival rate of 0.1% and a 1-year survival rate of 13.1%; numbers that raise red flags when compared with survival rates worldwide. The most recent 5-year survival rate in the US^35,39^ and in wealthier countries in the Middle East and North Africa region (commonly referred to as MENA) (eg, Qatar)^40^ approximated 25%. Our reported survival rates are notably lower than those observed in low-resource countries almost 2 decades ago (8.9%)^41^ and in neighboring nations facing political and financial challenges, such as Jordan (approximately 10%).^42^ Our analysis also illustrates an important association between this poor survival rate and armed conflicts by showing significantly poorer outcomes for patients who resided in regions that were classified relatively more insecure in the period of their presentation. It is important to note that this difference is not matched by higher disease stages or different demographic characteristics, as the regression analysis showed that the geographic-temporal distribution of cases explained more of the variability in survival than the age of the patient and the stage of the disease combined. Therefore, the poor outcomes in patients with lung cancer in our settings might be attributed more to disrupted accessibility to oncological care, limited availability of some therapeutic options, and the financial challenges to access the available ones.^8^ Despite still being characterized as also having poor outcomes, patients with lung cancer among Syrian refugees in Turkey exhibited a surprisingly better survival rate of 1.4% at 57 months compared with our sample.^43^ While the poor survival rates in both populations may have been influenced by the aforementioned factors, the observed difference can be attributed to the relatively safer commuting conditions and greater accessibility to specialized care for Syrian refugees in Turkey. These war-attributed factors are in line with constraints reported in other parts of the world during war times, such as in Afghanistan and Gaza, where much of the diagnostic and therapeutic oncological services became inaccessible.^7^

Finally, cancer care represents a huge economic challenge for Syria and neighboring countries. This burden has been explicitly studied in countries receiving the highest number of Syrian refugees,^44^ and is expected to have a higher gravity in Syria. The dramatic collapse of the Syrian economy put most Syrians in the grip of hunger,^45^ and was additionally affected by the imposed sanctions and corruption as well as the loss of human resources,^23,38^ restricting the ability of cancer care to recover. Accordingly, the recovery of the Syrian health care system faces significant challenges in the absence of a full resolution of the war. Rebuilding destroyed facilities and enticing specialized oncologists who have fled the country to return necessitates decades-long plans. Nevertheless, online consultations from oncologists abroad to general practitioners in conflict areas can temporarily alleviate the situation.^8^ Additionally, nationwide campaigns to enhance patient awareness and compliance could potentially expedite diagnoses and mitigate the prevalent issue of high treatment abandonment rates,^43^ ultimately contributing to a relative improvement in the gravity of the situation for Syrians both within Syria and abroad.

### Limitations

This study had several limitations. There was significant loss of follow-up, which was inevitable due the highly dynamic living situation of the Syrian population during war (ie, high internal and external displacement). Additionally, our sample did not include patients in besieged area, who could not present to our center. Nevertheless, reports from oncologists in these areas describe similar or poorer scenarios,^8^ suggesting that our results might underestimate poor outcomes in these areas. Finally, the scanned medical archives reported patients’ place of residence at the first presentation. Therefore, we cannot rule out that a subset of our sample might have moved among regions during the follow-up.

## Conclusions

A considerable number of patients with lung cancer likely faced challenges in reaching available specialized cancer care during the war in Syria. Moreover, diagnosed cases during the conflict often presented at advanced stages, consistent with and potentially linked to delayed presentation or diagnosis. Survival probabilities during the war were significantly reduced in correlation with the strength of armed conflict in the region of residence.

## Notes

Supplement 1. **eFigure.** Survival, Death, and Loss of Follow-up at Each Follow-up Time Point **eTable 1.** Binary Regression Analysis: Exploring the Impact of Disease Stage and Age Groups on One-Year Survival in Patients With Lung Cancer **eTable 2.** Binary Regression Analysis: Exploring the Impact of the War-Related Tempo-Geographical Regions, Disease Stage, and Age Groups on One-Year Survival in Patients With Lung Cancer

Supplement 2.
Data Sharing Statement
