# Supplementary material for: Lung Cancer Diagnoses and Outcomes During the Syrian War, 2011-2018
Source: JAMA Netw Open. 2024 Mar 13;7(3):e242091. doi: 10.1001/jamanetworkopen.2024.2091 (PMC10938179; doi:10.1001/jamanetworkopen.2024.2091)
Supplement: Supplement 1. — eFigure. Survival, Death, and Loss of Follow-up at Each Follow-up Time Point eTable 1. Binary Regression Analysis: Exploring the Impact of Disease Stage and Age Groups on One-Year Survival in Patients With Lung Cancer eTable 2. Binary Regression Analysis: Exploring the Impact of the War-Related Tempo-Geographical Regions, Disease Stage, and Age Groups on One-Year Survival in Patients With Lung Cancer [file jamanetwopen-e242091-s001.pdf]

Supplemental Online Content

Hanafi I, Abo Samra D, Alsaqqa R, Naeem A, Shebli B, Ajlyakin G. Lung cancer diagnoses and outcomes during the Syrian War (2011-2018). *JAMA Netw Open.* 2024;7(3):e242091. doi:10.1001/jamanetworkopen.2024.2091

eTable 1. Binary Regression Analysis: Exploring the Impact of Disease Stage and Age Groups on One-Year Survival in Patients With Lung Cancer

eTable 2. Binary Regression Analysis: Exploring the Impact of the War-Related Tempo-Geographical Regions, Disease Stage, and Age Groups on One-Year Survival in Patients With Lung Cancer

eFigure. Survival, Death, and Loss of Follow-up at Each Follow-up Time Point

This supplemental material has been provided by the authors to give readers additional information about their work.

© 2024 Hanafi I et al. *JAMA Network Open.*

eTable 1. Binary Regression Analysis: Exploring the Impact of disease stage and age groups on one-year survival in lung cancer patients.

Variables	B	Sig.	Exp(B)	CI lower limit	CI upper limit
Stage		<.001			
II	-.904	.055	.405	.161	1.019
IIIa	-.890	.035	.411	.180	.939
IIIb	-1.470	<.001	.230	.099	.535
IV	-1.706	<.001	.182	.082	.404
Age groups		.012			
50-59	-.331	.048	.718	.517	.997
60-69	-.467	.005	.627	.452	.870
>70	-.605	.004	.546	.363	.820
Constant	.005	.991	1.005		

Categories for reference include disease stage I and age group under 50.

© 2024 Hanafi I et al. *JAMA Network Open.*

eTable 2. Binary regression analysis: exploring the impact of the war-related tempo-geographical regions, disease stage, and age groups on one-year survival in lung cancer patients.

Variables	B	Sig.	Exp(B)	CI lower limit	CI upper limit
Region*Diagnosis year		<.001			
Eastern by 2012	-1.573	.003	.207	.075	.576
Eastern by 2013	.882	.001	2.416	1.413	4.133
Eastern by 2014	-.237	.548	.789	.365	1.708
Eastern by 2015	-.602	.145	.548	.244	1.231
Eastern by 2016	-1.632	.026	.196	.047	.822
Eastern by 2017	.060	.880	1.062	.485	2.323
Eastern by 2018	-19.641	.998	.000	-	-
Northern by 2012	-19.388	.997	.000	-	-
Northern by 2013	.392	.259	1.480	.750	2.921
Northern by 2014	-.061	.873	.941	.446	1.985
Northern by 2015	-1.360	.025	.257	.078	.846
Northern by 2016	-.962	.115	.382	.116	1.262
Northern by 2017	1.077	.002	2.935	1.468	5.868
Northern by 2018	-.623	.563	.536	.065	4.418
Southern by 2012	-1.097	.075	.334	.100	1.118
Southern by 2013	-.465	.346	.628	.239	1.653
Southern by 2014	.356	.309	1.428	.719	2.835
Southern by 2015	-.794	.104	.452	.174	1.177
Southern by 2016	-.077	.878	.926	.349	2.461
Southern by 2017	.039	.937	1.040	.391	2.768
Southern by 2018	-.171	.873	.843	.103	6.863
Western by 2012	-2.088	<.001	.124	.038	.399
Western by 2013	-.064	.872	.938	.428	2.053
Western by 2014	-.524	.235	.592	.249	1.407
Western by 2015	-.730	.085	.482	.210	1.107
Western by 2016	-.385	.329	.680	.314	1.476
Western by 2017	.594	.040	1.810	1.027	3.191
Western by 2018	.129	.842	1.138	.320	4.042
Stage		<.001			
II	-1.044	.040	.352	.130	.954
IIIa	-1.152	.012	.316	.128	.779
IIIb	-1.782	<.001	.168	.067	.424
IV	-2.023	<.001	.132	.055	.317
Age groups		.006			
50-59	-.396	.023	.673	.479	.946
60-69	-.525	.003	.591	.420	.833
>70	-.671	.002	.511	.336	.779

© 2024 Hanafi I et al. *JAMA Network Open.*

Constant	.532	.242	1.702		

Reference categories encompass disease stage I, age group under 50, and diagnoses that occurred in 2011 and in the central region.

© 2024 Hanafi I et al. *JAMA Network Open.*

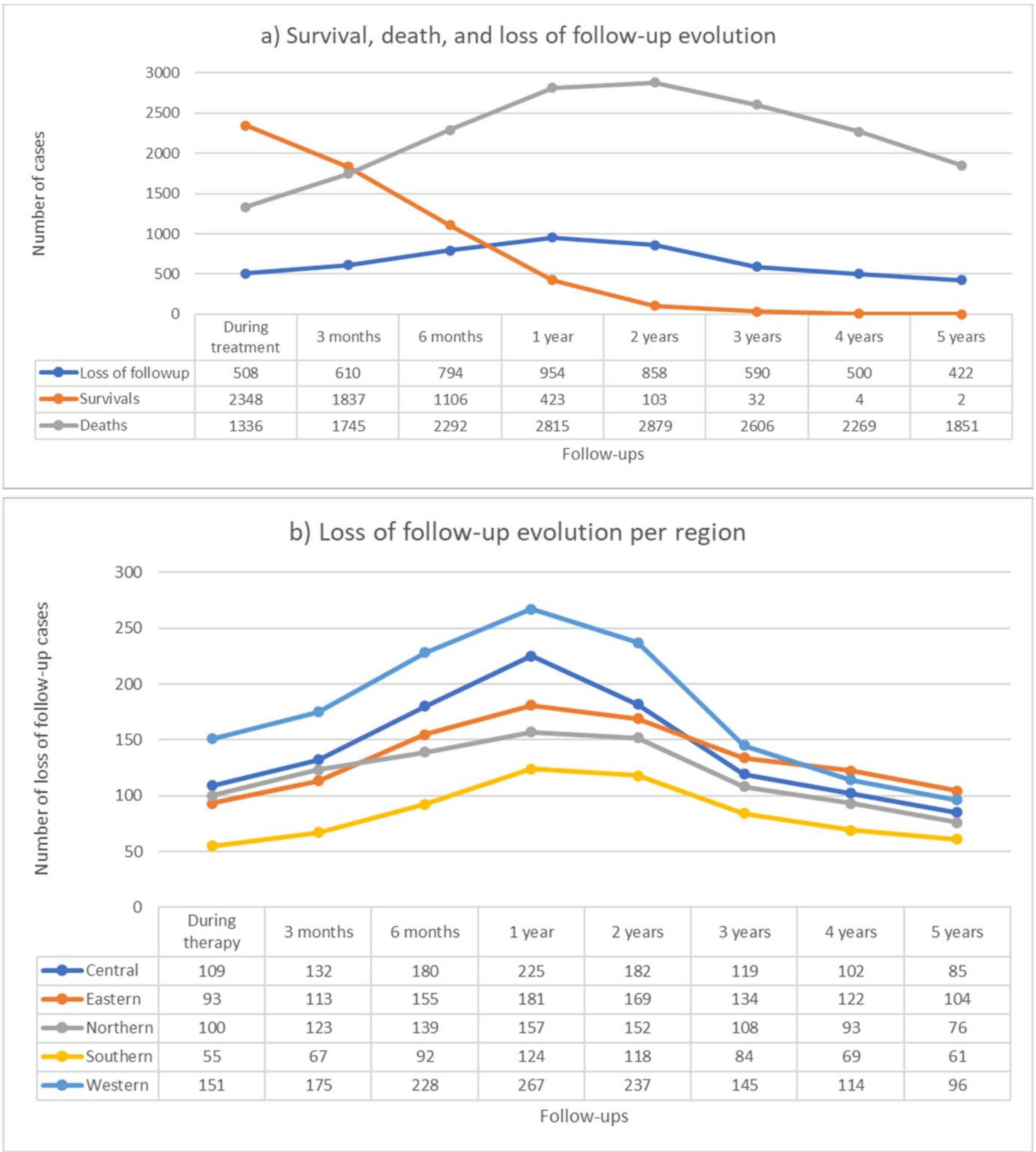

eFigure. Survival, death, and loss of follow-up at each follow-up timepoint.

A line chart depicting the numbers of loss of follow-up cases in the whole sample (a) and organized in geographic regions (b).

© 2024 Hanafi I et al. *JAMA Network Open.*
